# Comparison of Skinfold Thickness Measured by Caliper and Ultrasound Scanner in Normative Weight Women

**DOI:** 10.3390/ijerph192316230

**Published:** 2022-12-04

**Authors:** Zdzisław Lewandowski, Ewelina Dychała, Agnieszka Pisula-Lewandowska, Dariusz P. Danel

**Affiliations:** 1 Department of Human Biology, Faculty of Physiotherapy, Wroclaw University of Health and Sport Sciences, 51-612 Wroclaw, Poland; 2 Department of Fundamentals of Cosmetology, Faculty of Physiotherapy, Wroclaw University of Health and Sport Sciences, 51-612 Wroclaw, Poland; 3 Department of Anthropology, Hirszfeld Institute of Immunology and Experimental Therapy Polish Academy of Sciences, ul. Rudolfa Weigla 12, 53-144 Wroclaw, Poland

**Keywords:** adipose tissue, caliper, ultrasound scanner, adiposity

## Abstract

Obesity is a major issue affecting not only adults but also children in many places of the world. There are numerous methods for estimating the body fat percentage, however, all of those methods are different in terms of availability, accuracy, and the cost of an individual examination. The aim of this study was to compare two relatively easy and widespread measurement methods for assessing skinfold thickness: the BodyMetrix BX2000 ultrasound machine and a classic GPM caliper. Fifty-eight young women aged 19–24 years with normative body weight participated in the study. We found that although the measurements performed by both methods are positively correlated, the obtained values were different. In seven out of nine measured points, these differences were statistically significant. The measurements of skin fat folds with a caliper showed a higher value of subcutaneous tissue compared to ultrasound measurements. Only the values of measurements on the pectoral and mid-axillary did not differ between the methods. We conclude that due to the significant discrepancies in the values of measured skinfold thickness, appropriate measurement tools and dedicated formulas estimating the amount of body fat should be used.

## 1. Introduction

As a lifestyle disease [1,2], obesity is a major issue that affects not only adults, but also children. The excessively accumulating adipose tissue strains each system and organ in the human body. The health-related consequences thereof severely impair the functioning of the body. Therefore, measuring body fat is essential in the evaluation of general health status, unhealthy diet and eating habits, certain types of hormonal imbalance, training, and maintenance of healthy body weight. There are numerous methods for estimating the body fat percentage, such as anthropometric measurements, hydrodensitometry, BIA (Bioelectrical Impedance Analysis), DEXA (Dual-Energy X-ray Absorption), TBK (Total Body Potassium), CT (Computed Tomography), MRI (Magnetic Resonance Imaging), or US (ultrasonography). However, all those methods differ regarding availability, accuracy, and cost of an individual examination. Some of the most available methods [3] for estimating the body fat percentage and, thus, the body composition are the anthropometric measurements. The body fat percentage can be estimated based on the data obtained from skinfold thickness measurements and by using formulas that also take into account gender, age, body mass, and body height. Those formulas include the Slaughter equation, the Deurenberg equation, Lean’s equation, as well as the Jackson-Pollock three-site skinfold formula and the Durnin-Womersley four-site skinfold formula [4,5,6,7].

Furthermore, a local adipose tissue measurement by calipers gives insight into its distribution, which may prove to be a more valuable item of data than the total fat mass in the body. For instance, the accumulation of adipose tissue in the gluteal-femoral region (typical for women) causes less serious health-related consequences than the visceral adiposity (typical for men) where the adipose tissue deposits tend to be located in the abdominal region. This is because the human torso contains crucial organs, such as the heart, the liver, and the kidneys and the more adipose tissue around those organs, the greater the strain placed on them, the poorer their performance and the higher the risk of cardiovascular disease, diabetes mellitus, sleep apnea, hypertension, gallbladder disease, and many other conditions [8,9,10,11,12,13].

A caliper is an instrument used for measuring skinfold thickness that allows the assessment of the thickness of the subcutaneous adipose tissue [14]. The main advantage of the skinfold thickness measurement is that the caliper is a small, uncomplicated and low-cost instrument that can be conveniently taken and used anywhere. It provides a quick measurement procedure that shows whether the patient suffers from excessive accumulation of the subcutaneous adipose tissue [15]. However, this method is not without its flaws. The measurements may slightly differ from one another due to the skinfold-grasping technique, the site, and the positioning angle of the caliper. Therefore, to eliminate potential discrepancies, it is recommended to take several measurements and calculate the average result, as well as ensure that all patients’ measurements are taken by the same person.

Another method for assessing the thickness of the adipose tissue is ultrasonography. The ultrasound scanner is a device equipped with an ultrasound-emitting transducer that, once connected to a computer with appropriate software installed, allows the assessment of the tissues’ thickness. If the signal emitted by the transducer is reflected and returns to the transducer, it can be used to produce an image on the monitor [15]. Due to their properties (reflection or dispersion), ultrasonic waves can be used to produce images of various human tissues. Such measurements should be taken with the use of ultrasound gel that facilitates the transmission of ultrasounds and the movement of the transducer on the patient’s skin.

Compared to calipers, the quality of ultrasound results is much more prone to measurement methodology and device specifications. The devices might differ from one another in terms of many parameters, such as the angular and temporal resolutions, the focusing, the transducer used, the methods for preliminary and secondary processing, the signal enhancers and even the presentation of results, as well as many other parameters. Practical device operation skills and proper training before the examination are also of importance—dedicated sets of rules and guidelines and standard operating procedures have been developed and should be applied to various body regions so that the results could be comparable and useful in the medical diagnostics [16].

As mentioned above, both anthropometric and ultrasonography are relatively widespread methods in assessing body fat. Due to the methodological aspects, however, both methods may potentially yield different results and studies comparing both methods are scarce. Therefore, the current study aims to compare two measurement methods for assessing skinfold thickness using the BodyMetrix BX 2000 by IntelaMetrix (Livermore, CA, USA) ultrasound machine and a classic GPM caliper.

## 2. Materials and Methods

The sample group consisted of 58 young adult women (aged 19–24), students of the Wroclaw University of Health and Sport Sciences. The average body weight of the subjects amounted to 58.2 kg (SD 5.1), and their BMI (kg/m^2^) fell within the range of 18.5 to 24.9, with an average of 21.3 (SD 1.8).

The measurements of the adipose tissue thickness with an ultrasound scanner and the skinfold thickness with a GPM caliper were taken by the same person twice at all examined sites, on the right-hand side of the body and with the patient standing. The average result from both measurements was used for statistical analyses. The technical error of measurement was calculated according to Perini [17], and results are presented in Table 1.

The skinfold thickness was measured using a standard measuring protocol following Martin-Saller’s method [18] closely. We used a Harpenden-type caliper, with a constant tissue force of 10 g/mm^2^. Before each measurement, it was ensured that the caliper pointer was calibrated to the zero position. The measurer grasped a skinfold by the thumb and index finger of one hand and gently pulled it away from the body. Holding the caliper in the other hand, the measurer read the result approximately 3 s after the jaws of the instrument on the skin fold had been opened. The thickness of the adipose tissue was measured using the BodyMetrix BX2000 Intelametrix, a commercially available portable ultrasound scanner for non-medical use. Before taking measurements, the device was calibrated in accordance with the manufacturer’s instructions. The transducer was placed at the measurement site with the ultrasound gel applied, and the result was read using the software supplied by the manufacturer of the device.

Both the caliper and ultrasound scanner were used to measure the following anatomical sites:

- Triceps skinfold—a vertical fold located in the back of the arm, at mid-height, on the triceps;
- Biceps skinfold—a vertical fold located at the mid-height of the arm, in the front, on the biceps;
- Subscapular skinfold—at the inferior angle of the scapula, 2 cm below and at a 45° angle in parallel to the interior angle of the scapula;
- Mid-axillary skinfold—a horizontal fold on the side of the torso directly above the iliac crest, at the tenth rib;
- Pectoral skinfold—a diagonal fold approx. 1/3 of the way from the nipple to the armpit, closer to the armpit;
- Abdominal skinfold—a diagonal fold 1/4 of the way from the umbilicus to the anterior iliac spine;
- Supra-iliac skinfold—on the side of the body, 1–2 cm above the wing of ilium and at an approx. 45° angle from the body;
- Mid-thigh skinfold—a vertical fold in the front of the thigh, mid-way between the inguinal crease and the upper portion of the knee joint;
- Calf skinfold—a vertical fold below the popliteal fossa in the back of the calf, measured with the knee slightly flexed.

The measurements were taken at the Wroclaw University of Health and Sport Sciences in March 2017. All subjects gave informed consent to participate in the study.

### Statistical Methods

The Shapiro-Wilk test was used to verify whether the collected data followed a normal distribution. The analysis showed that most data deviated considerably from the normal distribution. Therefore, non-parametric methods were used in the study. In order to assess the correlation between the thickness of the adipose tissue measured in all women using the ultrasound scanner and the caliper (separate measurements for each site), Spearman’s rank correlation coefficient was used. However, the correlation assessment itself does not indicate statistically significant differences between the values of the analyzed variables. The Wilcoxon matched-pairs test was used to establish any statistically significant differences between the thickness of the adipose tissue at individual sites measured with the ultrasound scanner or the caliper. The analysis was carried out using STATISTICA (data analysis software system, www.statsoft.com accessed on 12 May 2017), ver. 13.1.

## 3. Results

Table 1 shows the measurement results and the descriptive statistics for all sites measured using the ultrasound scanner and the skinfold caliper.

The main analysis (Table 2) showed that in most of the measurement sites, the values obtained with both methods are statistically significantly positively correlated, except for three sites, i.e., biceps, middle axillary and abdominal, where no statistically significant correlations between the measurement were found.

Statistically significant differences between the thickness of subcutaneous adipose tissue measured with ultrasound and calipers were observed at 7 measuring points. At the triceps, biceps, subscapular, supra-iliac, mid-thigh, and calf sites, the measurements taken using the caliper were statistically significantly higher than the results of the measurements taken using the ultrasound scanner. In the case of the abdominal skinfold, the thickness values of the adipose tissue measured using the ultrasound scanner were statistically significantly higher than those obtained using the caliper. There were no statistically significant differences between the measurements of the pectoral skinfold and mid-axillary skinfold. Detailed results are presented in Table 2.

## 4. Discussion

In the current study, we compared measurements of the subcutaneous adipose tissue made by standard anthropometric caliper and ultrasound scanner. At most sites, significantly higher measurement values were obtained using the caliper. This is likely caused by the difference between the measurement methods. While using the caliper, the measurer grasps the fold along with a “double” layer of the skin, whereas in the case of the ultrasound scanner, the ultrasonic waves only need to penetrate a “single” layer of the skin. The two exceptions were the result of the measurement of the abdominal and pectoral skinfold where the value obtained using the ultrasound scanner was higher. While this observation for pectoral skinfold is difficult to explain, at least for the abdominal skinfold, the observed difference might be due to the distribution of the subcutaneous and visceral adipose tissues, the boundary between which is less pronounced. This observation is in line with the results from previous studies. Akyer et al. [19] also demonstrated higher results of the skinfold thickness measurements taken using a caliper, as well as a very high correlation between the results of the calf skinfold measurements.

The measurement values of subcutaneous adipose tissue obtained by the two methods were (in most cases) significantly positively correlated. However, the correlation coefficient values differed depending on the measurement site. Despite significant differences between the obtained values, the greatest correlations between were observed for the triceps skinfold (r = 0.7) and the calf skinfold (r = 0.6), which demonstrates that although both methods produce different final measurement results, they illustrate the distribution of the tissue at the measurement sites in a comparable manner. The lowest and insignificant correlation coefficient values were observed for the abdominal skinfold and the biceps skinfold (r = 0.1), which indicates that the measurements taken with the use of those methods could be the most scattered and inconsistent. The extreme values of the measurements also support this observation. It is also worth noting that from a methodological perspective, the most desirable situation is when measurement values obtained by both methods do not significantly differ and are highly positively correlated. This may indicate that both measurement methods are equivalent. However, in our study, such a situation was observed only for the pectoral skinfold. On the other hand, a different situation is when the measurement values differ significantly and are not correlated. In our study, this was observed for abdominal and biceps skinfold and may indicate that the two methods give divergent results.

Several authors also analyzed potential differences between anthropometric and ultrasound body fat measurements. Neves et al. [20] compared various methods for measuring adipose tissue thickness. However, the discrepancies between the results obtained using the ultrasound scanner and the caliper were different than those observed in the present study. In the Neves et al. [20] study, the measurements taken on 195 men divided into two groups (below and above the BMI of 25) using the ultrasound scanner produced higher results in 9 out of 12 measured skinfolds. The skinfold thickness was higher in triceps, biceps, pectoral, subscapular, mid-thigh, calf, mid-axillary, abdominal, and supra-iliac. The lower values were observed in subscapular, mid-thigh, and supra-iliac skinfolds.

According to Neves et al. [20], the differences between the greatest and the lowest correlation coefficient result from the anatomical differences are between calf and thigh. Since the calf is smaller than the thigh, so is the space between the muscle and the skin where the adipose tissue can be accumulated. Consequently, the precision of the measurements taken by different methods from relatively distinct structures may be different, resulting in differences between correlation coefficients also observed in our study.

While discussing our results, it should be noted that even by employing the same measurement method, the results may differ.

The differences may stem from different brands of instruments as well as different measurement techniques applied in each study. Ultrasound scanners and calipers manufactured by various companies may differ in construction or ultrasound software, which may cause discrepancies between the measurement results. For instance, Cyrino et al. [21] noted a discrepancy of 5.2% to 6.9% between the measurements obtained with various calipers. Furthermore, the results may also be biased by certain discrepancies between the precise measurement sites and slight differences in the skinfold-grasping techniques employed by different researchers. However, in our study, all measurements were made by the same person using the same instruments, minimizing potential biases in the observed results.

Nonetheless it is also worth noting that the applied adipose tissue measurement methods are characterized by certain limitations and measurement errors. According to Wagner [22], while having many advantages, ultrasonography is not a perfect imaging technique. For instance, artifacts can cause a fascia to be erroneously interpreted as adipose tissue. Toomey et al. [23] presented the discrepancies between the results of the skinfold thickness measurements that resulted from diverse pressure exerted by the ultrasound transducer on the examined tissues. If the transducer was applied to the given measurement site with excessive force, the results of the adipose tissue thickness measurement at that site were lower. Likewise, minimum pressure caused the results to be much higher. Therefore, the measurements should be ideally taken by one person with previous experience performing this type of examination.

One potential limitation of our findings may be related to the specific character of our sample. In the current study, we examined only young adult women in a relatively narrow range of body mass index (and adiposity). Future research would benefit from including larger groups of men and women (with sex differences in the adipose tissue distribution) at various stages of obesity, as well as from different ethnic groups.

Moreover, using multiple ultrasound scanners and various skinfold calipers would allow further detailed analysis of the potential discrepancies between the results. In our study, the TEM values for USG measurements were generally higher than for SC, indicating that the USG method was less accurate. Nonetheless, the USG scanner used in the study was a non-medical device that, according to the manufacturer, could be operated even by inexperienced users. Using trained and experienced sonographers, however, may greatly reduce measurement errors (cf. [24]). Therefore, results from future large-scale studies involving various devices may help develop standard practices to be followed while using specific measurement methods. That in turn would enable the researchers to compare the results obtained at various centers more accurately.

## 5. Conclusions

The results from our study show that the measurements taken using the ultrasound scanner and standard anthropometric caliper can vary. Observed discrepancies imply that while calculating, for instance, the body fat percentage based on the thickness of the adipose tissue, the measurement tools (equations, algorithms) developed specifically for the given estimation method should be used.

The results suggest that the methods are not identical (although results are often significantly positively correlated, their values may be different) and that using various measurement tools may lead to discrepancies.

Consequently, the results obtained while applying those methods cannot be used interchangeably in direct comparisons and during the interpretation of the subjects’ adiposity.

## Figures and Tables

**Table 1:** The thickness of the subcutaneous adipose tissue in the study sample (*n* = 58 women). USG represents a measurement taken using the ultrasound scanner; SC represents a measurement taken using the skinfold caliper. Q1 and Q2—quartiles 1 and 2, respectively. TEM—technical error of measurement.

Measure	Method	Average [mm]	SD [mm]	Q1	Median [mm]	Q3	Min. [mm]	Max. [mm]	Absolute TEM	Relative TEM (%)
triceps skinfold	SC	15.9	3.6	13.5	15.2	17.8	8.0	25.9	0.75	4.69
	USG	10.0	2.6	8.1	9.5	12.0	4.8	15.9	0.73	7.28
biceps skinfold	SC	10.3	3.5	7.4	10.0	13.0	5.1	18.1	1.23	11.91
	USG	7.4	3.5	5.0	6.8	8.6	3.1	21.5	2.3	31.07
subscapular skinfold	SC	10.8	3.0	8.8	10.1	11.4	6.3	24.1	0.36	3.31
	USG	7.6	1.9	6.7	7.4	8.3	3.7	12.8	1.33	17.49
mid-axillary skinfold	SC	8.5	2.7	6.3	7.4	10.6	4.9	14.3	0.38	4.46
	USG	8.5	1.9	7.4	8.4	9.8	4.4	12.4	1.13	13.33
pectoral skinfold	SC	6.9	2.5	5.1	6.0	8.2	4.0	14.0	0.61	8.85
	USG	7.5	2.8	5.5	7.1	8.8	2.9	19.0	1.09	14.58
abdominal skinfold	SC	14.0	5.0	11.1	13.5	15.6	6.5	41.2	3.22	23.0
	USG	21.5	6.7	16.4	22.2	26.2	6.6	36.3	2.9	13.48
supra-iliac skinfold	SC	11.4	4.0	8.4	10.5	14.1	4.2	27.9	0.95	8.39
	USG	7.8	2.5	5.8	7.8	9.3	3.3	13.5	1.16	14.81
mid-thigh skinfold	SC	23.4	4.7	19.5	23.0	27.5	13.2	33.9	1.18	5.04
	USG	9.9	2.2	8.6	9.5	11.0	5.2	15.7	0.83	8.41
calf skinfold	SC	8.1	2.8	6.2	7.3	9.3	4.1	20.0	0.49	5.97
	USG	7.2	1.9	5.9	7.2	8.6	3.9	12.6	1.23	16.99

**Table 2:** Correlations between the measurements of the adipose tissue thickness obtained using the USG and the SC and the statistical significance of the differences between the measurement obtained from both methods (*n* = 58).

Site	Correlations between Measurements		Differences between Measurements	
	R	*p*	Z	*p*
triceps skinfold	0.7	<0.05	6.5	0.0001
biceps skinfold	0.1	>0.05	4.1	0.0001
subscapular skinfold	0.5	<0.05	5.9	0.0001
mid-axillary skinfold	0.2	>0.05	0.4	0.7131
pectoral skinfold	0.5	<0.05	0.6	0.5408
abdominal skinfold	0.1	>0.05	5.3	0.0001
supra-iliac skinfold	0.4	<0.05	5.7	0.0001
mid-thigh skinfold	0.5	<0.05	6.6	0.0001
calf skinfold	0.6	<0.05	2.9	0.0041

## Data Availability

Not applicable.
